# Causal Mapping of Bodily Awareness and Mesoscale Circuit Organization in the Human Cingulate and Precuneus

**DOI:** 10.21203/rs.3.rs-10503199/v1

**Published:** 2026-08-06

**Authors:** Dian Lyu, Lamara Allen, Sofia Pantis, Elif Goksun Karagoz, Julian Quabs, Josef Parvizi

**Affiliations:** 1. Department of Neurosurgery, The Second Affiliated Hospital, Zhejiang University School of Medicine, Hangzhou, China; 2. MOE Frontier Science Center For Brain Science and Brain-Machine Integration (BBMI), Zhejiang University, Hangzhou, China; 3. Laboratory of Behavioral and Cognitive Neuroscience, Stanford University School of Medicine, Palo Alto, California, USA; 4. Institute of Neuroscience and Medicine (INM-1), Research Centre Jülich, Jülich, Germany; 5. Department of Neurology & Neurological Sciences, Stanford University School of Medicine, Palo Alto, California, USA

**Keywords:** cingulate gyrus, insula, interoception, intracranial electrical stimulation, cerebro-cerebral evoked potentials, functional imaging, hemispheric asymmetry, lateralization, connectivity

## Abstract

How the human brain generates subjective bodily experience remains a fundamental question in cognitive neuroscience. The cingulate cortex and precuneus (CC/PCu) have been implicated in bodily awareness and self-related processing, yet the functional architecture and causal relevance of these regions and the circuit mechanisms supporting conscious bodily states remain unclear. Here, we combined intracranial electrical stimulation, first-person reports, causal electrophysiological connectivity mapping and large-scale functional network analyses to investigate the human CC/PCu in 63 individuals. Across 660 stimulation sites, we identified a mesoscale functional mosaic in which the stimulation of neighboring cortical populations, separated by millimeters, gave rise to categorically distinct subjective experiences, ranging from localized sensorimotor sensations to complex integrated bodily states. These phenomenological differences were not explained by anatomical location alone, but by distinct causal connectivity profiles. Sensorimotor-responsive sites preferentially exhibited divergent outgoing connectivity, whereas complex bodily sites showed convergent incoming connectivity from distributed networks. Among these pathways, connectivity between the CC/PCu and posterior insular cortex emerged as a critical determinant of whether local stimulation produced a reportable bodily experience: millimeters away, neighbouring sites lacking this connectivity remained silent. We further identified a right-lateralized network architecture supporting bodily experience across both neuroimaging and electrophysiological connectivity measures. Together, these findings reveal that conscious bodily states may be rooted in fine-grained mesoscale circuits embedded within large-scale brain networks, demonstrating that connectivity architecture, rather than anatomical location alone, determines the capacity of human cortical sites to be involved in conscious and subjective bodily awareness.

## Introduction

How the brain processes information about the body and generates awareness of its internal states that shape our emotions, decisions, sense of agency, and adaptive behavior remains among the most fundamental yet least understood questions in neuroscience. Converging evidence has implicated the cingulate cortex and precuneus (CC/PCu) as key components of a distributed cortical and subcortical system that supports this capacity for bodily self-awareness^1–16^. Dysfunction of CC/PCu regions has been consistently implicated in psychiatric conditions such as schizophrenia and depression that are marked by distorted or diminished self-experience^17–21^. Understanding the functional architecture through which the CC/PCu complex helps generate bodily states provides a crucial step towards explaining how these regions contribute to embodied subjective experience, while informing the rational development of future neurostimulation therapies targeting these circuits.

While neuroimaging studies have provided an extensive map of CC/PCu activity in the human brain, and animal studies have yielded important causal insights through direct experimental manipulation, each approach has inherent limitations when it comes to addressing questions of subjectivity. Neuroimaging studies in humans have provided important *correlative* evidence with limited causal inference, whereas animal studies, despite permitting direct experimental manipulation and having yielded invaluable causal insights into the circuit-level mechanisms underlying brain function, have not been able to explore subjective phenomenology directly, as first-person conscious experience in non-human animals remains beyond current empirical reach. In this regard, intracranial recordings in humans, combined with direct cortical stimulations, offers a rare opportunity to bridge these two traditions: it affords the causal leverage of perturbational methods while preserving access to first-person phenomenological report, and does so with high anatomic precision at the individual level.

Although rare, intracranial investigation of the human CC/PCu complex in neurosurgical patients has a long history and continues to be an active area of inquiry. Among the earliest contributions, Talairach and colleagues conducted a pioneering intracranial electrical stimulation (iES) study of the cingulate, and reported movements of the upper chest and lower neck that were sometimes accompanied by shifts in mood or level of consciousness, as well as autonomic changes including pupil dilation, facial flushing, increased heart rate, and elevated respiratory frequency^22^. Since then, several other studies have reported subjective responses spanning sensory, motor, affective, autonomic, language, visual, and vestibular domains when sites within the CC/PCu complex were electrically stimulated in awake human subjects^8,23–26^.

Building on the prior studies, which have largely examined stimulation-evoked responses in isolation, without systematic characterization of the underlying network dynamics or cross regional relationships within the same individual brains, we took a multimodal approach to analyze a large cohort (660 sites, 63 participants) of direct stimulations of the CC/PCu sites over the last 18 years of our practice. Crucially, rather than limiting our analysis to the evoked subjective experiences and their anatomical correlates alone, we additionally mapped the network-wide causal connectivity of each stimulated site by characterizing the electrophysiological signals propagated across all available cortical recording sites in response to electrical stimulations. This allowed us to link reported subjective experiences not only to the precise anatomical location of the stimulated site, but also to its brain-wide connectivity fingerprint.

Collectively, our findings reveal a mesoscale mosaic-like functional architecture in the human CC/PCu, in which neuronal populations separated by mere millimeters engage dissociable circuits and give rise to categorically distinct subjective experiences. Our findings advance our understanding of CC/PCu function in two important ways: first, by characterizing the brain-wide causal effects of spatially localized electrical stimulations; and second, by identifying directional connectivity motifs between the CC/PCu and insular cortices that predict and distinguish the phenomenological quality of stimulation-evoked bodily experiences.

## Results

We report the results of two complementary studies employing distinct stimulation protocols for different experimental objectives. **Study I** investigated the subjective experiences elicited by 50 Hz electrical stimulation of the cingulate cortex (CC) and precuneus (PCu) in 63 participants. **Study II** mapped the causal electrophysiological connectivity of the same CC/PCu sites using repeated single-pulse electrical stimulation in a subset of 28 participants.

### Subjective feelings induced by intracranial electrical stimulation

**Figure 1.**
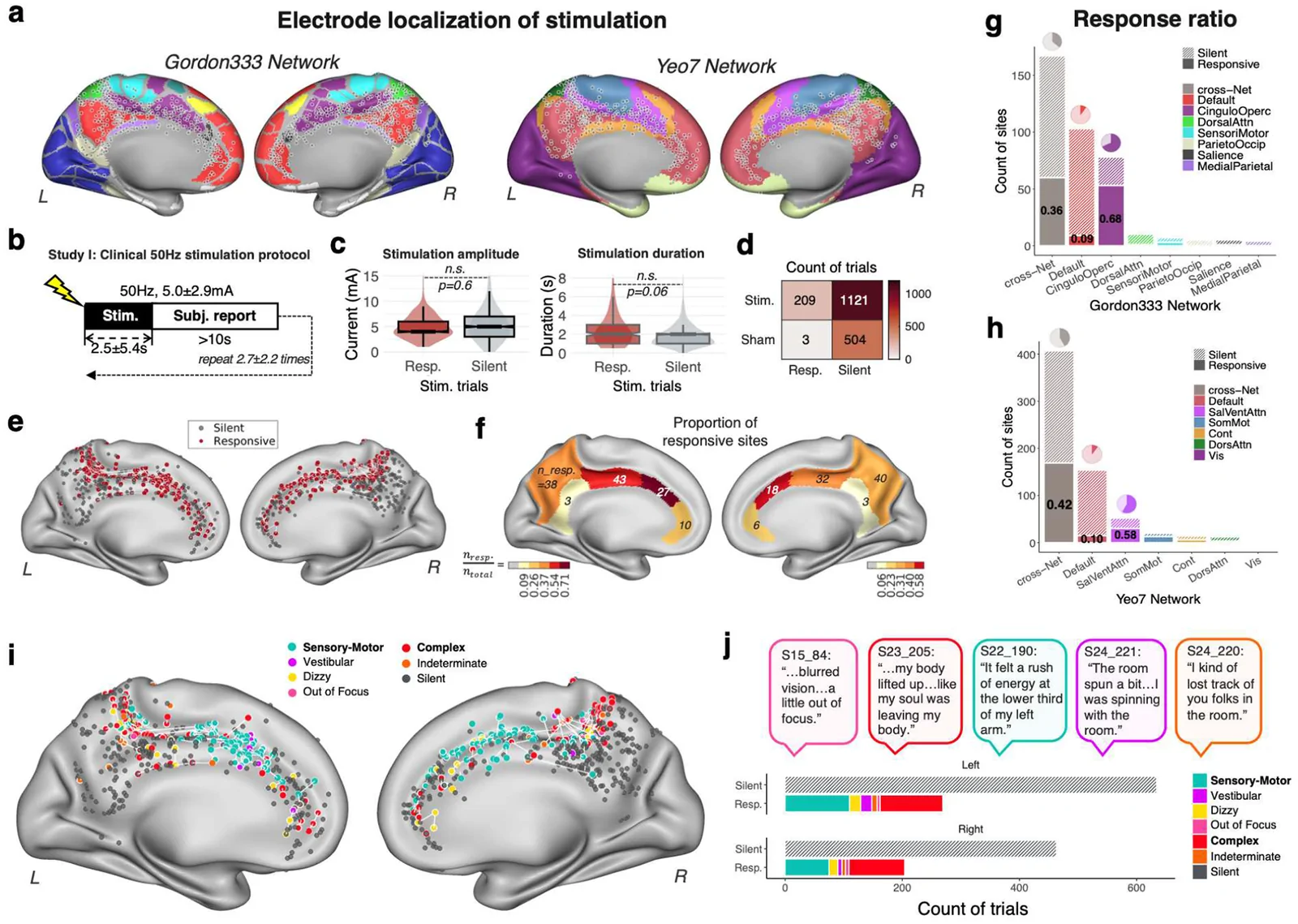
Subjective states induced by intracranial electrical stimulation of the cingulate and precuneus regions. **(a)** Each participant’s stimulated sites were localized and characterized in the native high-resolution T1 image. Only for visualization purpose, electrodes were registered to a standard brain surface space (i.e., FS_LR 32k template with homologous left and right hemispheres). Two intrinsic functional connectivity (FC) network atlases were superimposed to provide references of anatomical location of the electrodes (upper: Gordon333, lower: Yeo7)^31,32^. Electrodes were manually verified to be localized in the CC/PCu; apparent misalignment in the group-level visualization results from spatial normalization errors (see examples in Figure S3). **(b)** Standard 50Hz stimulation protocol was used to map functional responses. **(c)** Stimulation current and duration did not differ between Responsive and Silent trials. Standard box plots, superimposed with violin plots, illustrate basic statistics (1st, 2nd, and 3rd quantiles). **(d)** Counts of trials and reported subjective changes in the real vs. sham stimulations suggest that the placebo effect was rare. **(e)** Responsive (red) and silent (gray) stimulation locations on the bilateral brain surface are shown at the group level. **(f)** Ratios of responsive sites in different cingulate/precuneus segments are shown based on the Desikan-Killiany Atlas (https://surfer.nmr.mgh.harvard.edu/fswiki/CorticalParcellation). The ratios were calculated as the count of responsive sites divided by the total stimulation sites in each segment. Count of stimulation sites (responsive or silent) in each intrinsic FC network, using the Gordon333 **(g)** and Yeo7 **(h)** network atlases as references. **(i)** Stimulation locations for different categories of induced subjective changes are shown, including “Sensory-Motor”, “Vestibular”, “Dizzy”, “Out of Focus”, “Complex” and “Indeterminate.” Two electrode contacts linked by a white line indicate a bipolar pair of electrode contacts stimulated **(j)** A representative example of a subjective report is provided for each category, except for “Dizzy,” where participants literally reported experiencing dizziness. Trial counts of subjective reports in different categories and of silent trials are presented in the bar plot.

We collected data from 660 cingulate cortex (CC) and precuneus (PCu) stimulation sites across 63 participants (41.3% female, 96.7% right-handed; age: 37.9 ± 12.1 years). On average, 10.48 ± 8.27 CC/PCu sites were stimulated per participant (mean ± SD). Participant characteristics are provided in Supplementary Table S1. A small portion of the dataset have been previously published in two separate publications^8,27^. Stimulation sites were randomly distributed across cingulate/precuneus subregions without any apparent differences in the sampling density (Fig. 1a). We applied 50 Hz stimulation with a varied amplitude (5.0+/−2.9mA) and duration (2.5+/−5.4s) in a bipolar manner (between a pair of adjacent electrode contacts). As part of our standard quality assurance measures, we excluded data if subjective responses induced by stimulation (1) were reported during after-discharges or seizures, (2) were not reproducible with repeated stimulations of the same site, (3) or did not exhibit a clear dose–response relationship (i.e., effects increasing as stimulation intensity increased and vice versa), and (4) were also reported during sham trials, which were very rare (3 of 1837 trials), consistent with our prior report^28^.

As shown in Figure 1, we found that stimulation of specific anatomical sites within the CC/PCu regions reliably induced changes in the subjective state of the individual participants. We refer to sites inducing these subjective changes as “*responsive*” sites. We found the “responsive” sites to be distributed in a mosaic-like pattern, i.e., responsive and silent sites were juxtaposed to each other (Fig. 1f). Notably, the degree of responsiveness across various subregions– as defined by the Desikan-Killany atlas - varied significantly (χ^2^ = 5.80, df = 4, p < 0.001, Fig. 1g), without any difference between the two hemispheres (χ^2^ = 0.01, df = 4, p > 0.99). The “caudal anterior cingulate”, roughly corresponding to the anterior midcingulate in Vogt’s parcellation^29^, exhibited the highest proportion of responsive sites (left: 0.71; right: 0.58), whereas the isthmus subregion had the lowest (left: 0.09; right: 0.06).

Moreover, we found the responsiveness ratios to be different depending on the intrinsic functional networks spatially overlapped with the stimulated site. The mid-cingulate region within the action mode network (AMN) exhibited the highest response rates (0.68 in Gordon333; and 0.58 in Yeo7, where AMN roughly maps onto the salience/ventral attention network)^30–32^. Sites of default mode network (DMN) showed the lowest (0.09 in Gordon333; 0.10 in Yeo7), consistent with our prior studies^33,34^. Amplitude (p = 0.6) or duration (p = 0.06) of stimulations did not differ significantly between responsive and silent sites (Fig. 1c,d).

Careful review of bedside notes, validated against offline video transcriptions by independent documenters, showed that stimulation-induced subjective responses fell mainly into two broad categories: i. Sensory-Motor (SM, 15.61%) reflecting clearly distinguishable sensorimotor changes in a specific part of the body; and ii. Complex (CLX, 12.73%) reflecting more nuanced and integrated bodily experiences that could not be reduced to a single sensory or perceptual domain, such as depersonalization and dissociative sensations of floating in space following anterior precuneal stimulation (previously described as alterations in the sense of bodily self)^8^, and a sense of foreboding accompanied by changes in heart rate and sensations in the chest and neck while simultaneously feeling empowered to face impending challenges following anterior mid-cingulate stimulation (previously described as an induced will to persevere)^27^. We acknowledge that the CLX category was phenomenologically heterogeneous, and pooling all CLX reports for statistical comparisons may have obscured meaningful within-category differences. Nevertheless, the reported experiences consistently shared a common bodily dimension, despite not being reducible to simple bodily sensations. Because the aim of our analyses was to contrast these more elaborated bodily experiences with elementary somatomotor sensations, we considered the CLX category sufficiently coherent to be analyzed as a unified comparator.

Other less frequent instances of subjective reports were in the following domains: “dizzy” (3.48%) i.e., light headedness and nausea; “vestibular” (1.67%) i.e., spinning sensation; out of focus (0.76%) i.e., patients reporting their vision got blurry or they lost mental focus. In some instances responses were difficult to categorize either because the patient could not describe the nature of the change or the reports were un-interpretable due to technical issues (video quality, paucity of bedside notes). We labelled these as “Indeterminate” (1.52%). See reported word counts and word cloud for these categories in Supplemental (Suppl.) Figure S1.

### Lateralization of Subjective Responses

**Figure 3.**
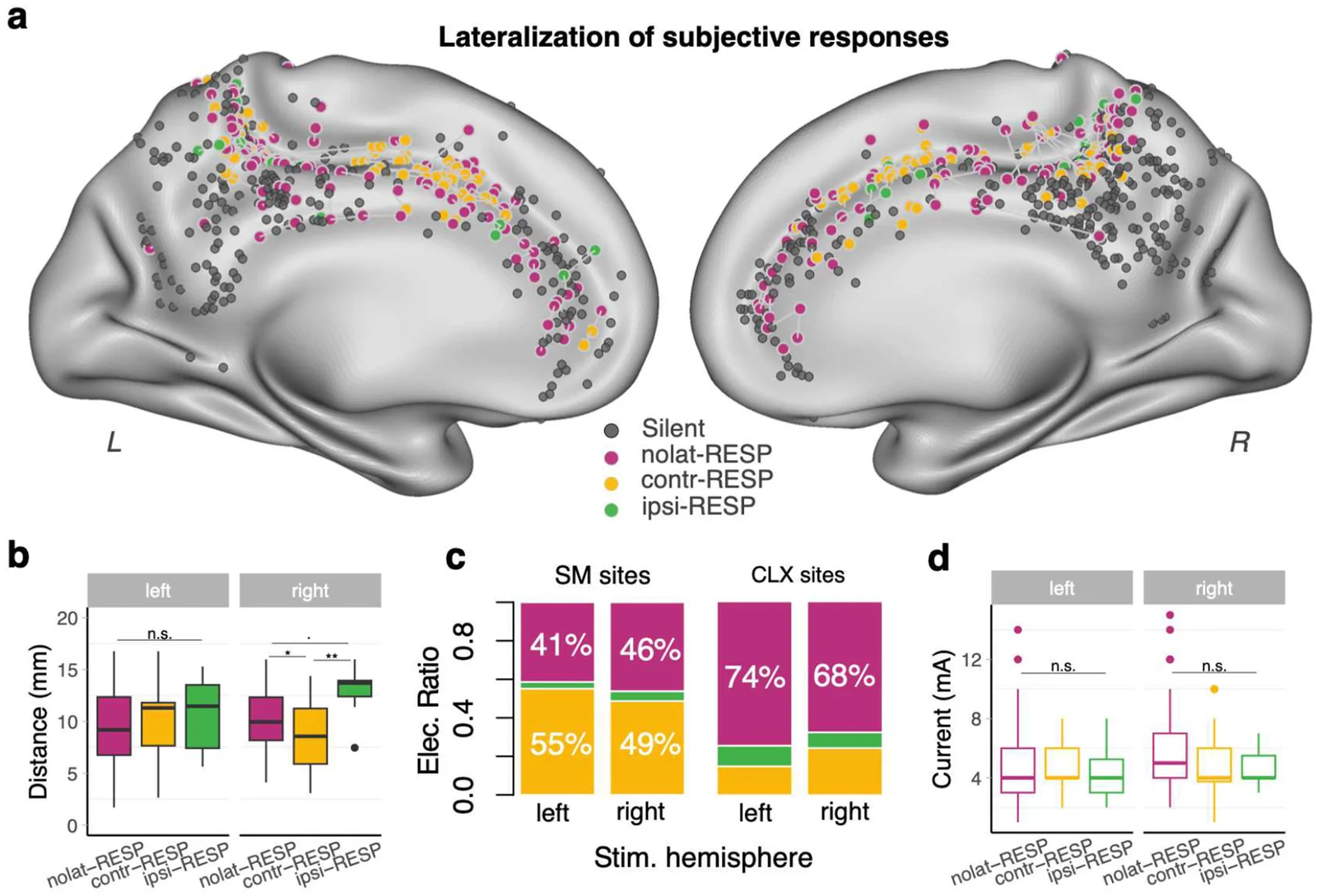
Laterality of Subjective Changes Induced by Cingulate/Precuneus Stimulation. (a) Stimulation locations on the bilateral brain surface that induced subjective changes of different laterality are shown, including ipsilateral responses (ipsi-RESP: subjective responses on the same side as the stimulation), contralateral responses (contr-RESP: responses on the opposite side of the stimulation), and responses without laterality (nolat-RESP). (b) Distance of the stimulation site to the mid-wall of the brain surface, used as a sanity check for the potential confounding effect of stimulation volume conduction spreading to the opposite hemisphere. This potential confounding effect was not significant. This measure is visualized using a standard box plot: the midline and edges of the box indicate the median, the first and third quartiles (Q1, 25th percentile), and individual outliers are plotted beyond the whiskers (i.e., 1.5 times the interquartile range). (c) Ratios of the responsive sites inducing bodily responses that are on the same side (ipsi-RESP), or opposite side (contr-RESP) of the stimulation, or not lateralized (nolat-RESP). (d) Current (mA) differences among the stimulation that induced bodily responses of different sides, visualized using a standard boxplot (same as Fig.3b).

We examined if the subjective responses following 50Hz stimulation of the CC/PCu regions were located ipsilateral/contralateral to the site of stimulation (ipsi/contr-RESP) or difficult to ascertain laterality (nolat-RESP). As detailed in Fig. 3, ipsilateral responses were rare and were not due to current spread to the contralateral hemisphere, as stimulation sites eliciting ipsilateral responses were neither located closer to the midline (Fig. 3b) nor stimulated with higher currents (Fig. 3d) than sites that did not elicit ipsilateral responses. As expected, CLX responses, which inherently reflect more integrated bodily information, yielded a higher proportion of non-lateralizable reports compared to SM responses. However, surprisingly, 40% of sensorimotor (SM) responses were also difficult to lateralize, involving either the body core or bilateral body representations. More details are presented in Suppl. Figure S2.

### Population-level connectivity profile of stimulated sites

**Fig. 4.**
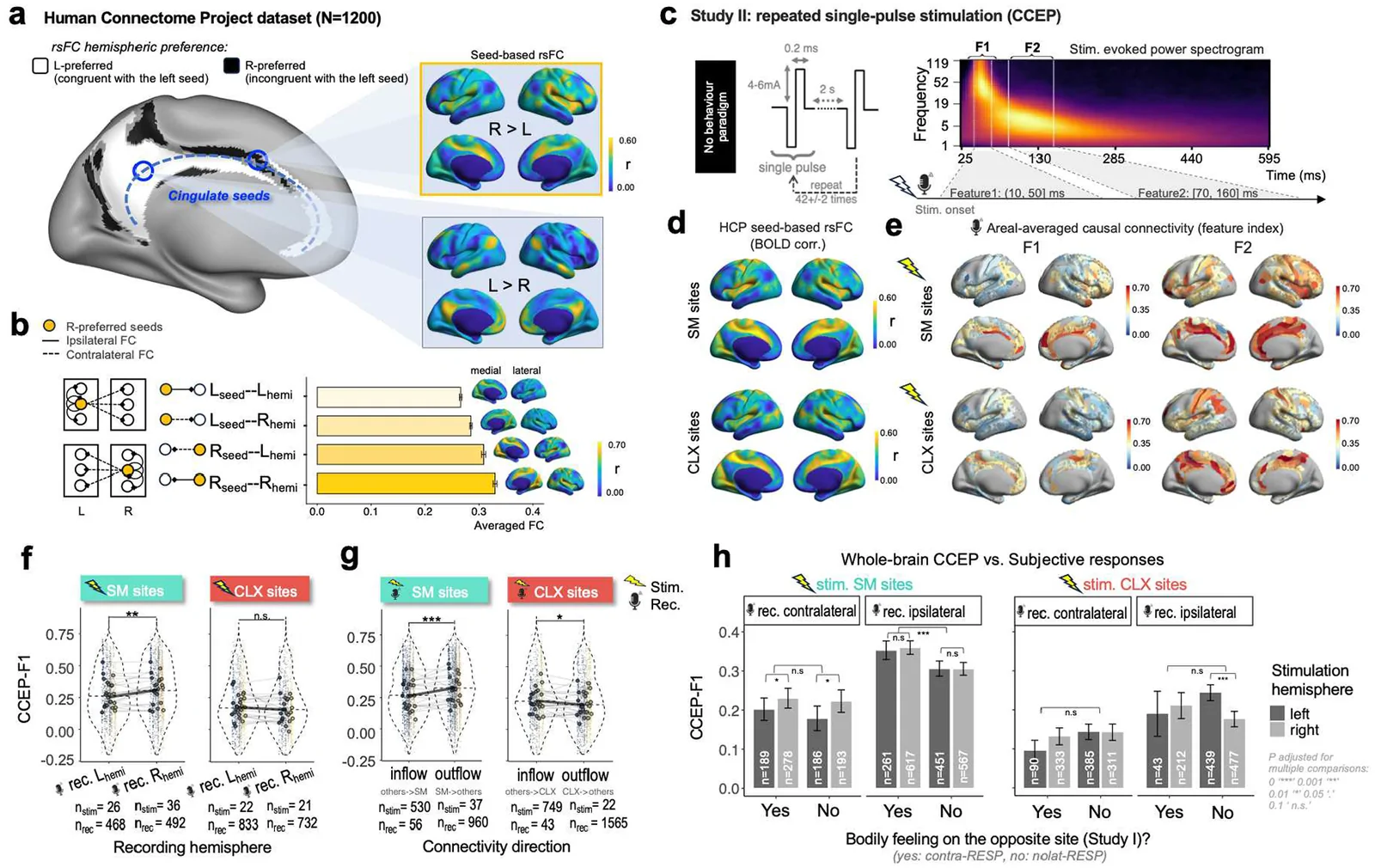
Connectivity Profiles of SM and CLX Responsive Sites. **(a)** While the right CC/PCu seeds show stronger ipsilateral functional connectivity (FC), specific areas in the *left* seeds (as shown in black) exhibited an unexpected preference of coupling to the *right* hemisphere. Hemispheric preference was determined by vertex-wise “left vs. right” paired t-tests (Bonferroni-corrected). Two examples are for right (up) and left (down) hemispheric preference. **(b)** Cross-hemispheric FC comparison for right-preferred (“R-preferred”) CC/PCu vertices (black in panel a) and their contralateral counterparts. Cartoon defines four FC categories (diamond end of an arrow denotes the seed’s coupling terminal; undirected). Adjacent medial/lateral views show hemisphere-specific FC (seed-to-vertex FC averaged across the seeds and vertices of one category); error bar: ±4 SE. **(c)** Schematic of the CCEP protocol during which repeated single-pulses (~42/site) were delivered to a pair of adjacent electrode contacts and then evoked responses were measured across all available recording sites in the brain. We measured F1 (early) and F2 (late) features as described in our recent publication^36^. These measures were extracted based on trial-averaged power (connection strength) and inter-trial phase coherence (connection stability). Group-level early (F1) and late (F2) features were presented in the spectrogram at the right. **(d)** Seed-based rsFC profiles for sensory-motor (SM) and complex (CLX) sites (localized in Study I, from both hemispheres) derived from Human Connectome Project data. **(e)** Whole-brain causal connectivity patterns induced by CC/PCu stimulation are visualized using areal-averaged F1 and F2 connectivity, computed by averaging connectivity across stimulation sites the stimulation within each responsive category and recording sites within each Glasser parcel (n_elec > 5). Electrode coverage is indicated by the grey dots on the brain surface. Within-regional connectivity comparisons (mixed linear model corrected for “subject:region” nested random effect) for testing hemispheric (left vs. right) and directional (outflow vs. inflow) differences. SM sites, regardless of the hemisphere in which they were stimulated, evoked stronger responses in the right hemisphere **(f)**, and SM sites overall had more outflow connections than inflow connections whereas this pattern was reversed for CLX sites **(g)**. Outflow refers to the strength of evoked responses caused in another region when a site is stimulated while inflow is the strength of evoked responses in the region of interest when another area was stimulated. Individual datapoints (small dots, colored by different brain areas) and areal averages (large dots) are shown. The horizontal dashed line in the violin plot indicates group median. **(h)** Group-level three-way interaction (stimulation hemisphere × response laterality × CCEP laterality) for SM and CLX sites. Error bar indicates deviation of two standard errors. Post-hoc comparisons are corrected. Sample sizes are indicated.

To probe the network mechanisms underlying the stimulation-induced subjective responses, we examined the connectivity profiles of the responsive cingulate sites – focusing only on the two most common SM and CLX sites. For this we used two modalities of connectivity measures: i) resting-state functional neuroimaging (i.e., BOLD signal correlation), and ii) causal electrophysiological connectivity measured by cerebro-cerebral evoked potentials (CCEP) that were derived from repeated single pulse stimulations (Fig. 4a, b).

Serving as contextual information, we conducted a seed-based resting-state functional connectivity (FC) analysis for all vertices in the CC/PCu regions using population-level database of the Human Connectome Project 210V dataset^35^. At least three main large-scale networks were identified using seeds in CC alone: the default mode network (DMN), the fronto-parietal control network (FPCN), and the action mode network (AMN) (see Suppl. Videos for network transitions along the posterior-to-anterior cingulate axis).

Significant FC laterality was observed in specific CC/PCu vertices. The laterality of FC for each seeding vertex was tested using a spatial paired t-test, which compared its functional connectivity across all vertices in the left hemisphere against its connectivity to the homologous vertices in the right hemisphere. The resulting p-values were then Bonferroni-corrected for the total number of seeds tested. Interestingly, this analysis revealed a significant preference of functional coupling to the right hemisphere in only specific regions that spatially overlapped with our responsive electrode sites (Fig. 4a).

Typically, FC of a cortical site is stronger with ipsilateral than contra-lateral cortical regions. However, seedings in some of the stimulated areas (shown as black vertices in Fig. 4a), revealed a distinct, right-biased connectivity that exhibited the following gradient: FC was weakest between left seeds and the left hemisphere, stronger between left seeds and the right hemisphere, even stronger still between right seeds and the left hemisphere, and strongest between right seed and right hemisphere. Overall, right-sided seeds exhibited higher global connectivity to both hemispheres than their left homologues, and the entire network was preferentially biased toward the right hemisphere (Fig. 4b). Notably, the spatial location of these regions and their seed-based FC patterns overlapped with the location of the medial subregions of the AMN (Fig. 4a)^30^

### Directional electrophysiological connectivity profiles of responsive sites

In Study II, we leveraged repeated single-pulse electrical stimulation to characterize the meso-scale causal electrophysiological connectivity of the responsive sites identified in Study I. Twenty-eight of the 63 participants from Study I underwent this procedure (42.9% female, 96.4% right-handed; mean age, 38.3 ± 11.7 years, see Supplementary Table S1 for cohort overlap). These participants contributed, on average, 71.43 ± 24.52 stimulation channels, 147.04 ± 10.51 recording channels, and 10,768.04 ± 4,606.09 directed stimulation–recording pairs per person, yielding a total of 301,505 stimulation–recording pairs spanning the whole brain. We quantified both the i) strength and ii) inter-trial coherence of the cerebro-cerebral evoked potentials (CCEPs) recorded from connected regions (see Methods and our recent methodological publication for details)^36^. Consistent with our previous work, spectral decomposition of the evoked responses revealed an early component (F1) and a delayed component (F2), reflecting feedforward and indirect connectivity, respectively (Fig. 4c)^36,37^. Because F2 largely amplified the spatial pattern observed in F1 without qualitatively altering the results, subsequent analyses focused exclusively on F1 (Fig. 4e).

The resulting electrophysiological connectivity data, derived from the same participants included in Study I, confirmed the right-preferred connectivity pattern observed in the HCP fMRI cohort (Fig. 4c,e,f). Importantly, the directional information uniquely provided by CCEPs allowed us to separately examine outflow connectivity (from the CC/PCu to other regions) and inflow connectivity (from other regions to the CC/PCu) using the same pairs of cortical sites within individuals. A nested random effect of brain region within participant was included to account for within-participant, within-region variability during left– right hemisphere comparisons. For SM sites, significantly stronger connectivity with the right hemisphere than the left hemisphere was observed for both outflow and inflow connections, regardless of the stimulated hemisphere (Fig. 4e; Linear Mixed Model [LMM]: n_conn_=2353, ΔF1=0.03, t=3.54, CI=[0.01, 0.05], p-adj.=0.003 for outflow; n_conn_=2032, ΔF1=0.05, t=5.24, CI=[0.03, 0.07], p-adj.=1.4e-06 for inflow). In contrast, CLX sites showed hemispheric lateralization only for inflow connectivity, which was likewise biased toward the right hemisphere (n_conn_=1495, ΔF1=0.04, t=3.27, CI=[0.01, 0.06], p-adj.=0.007, see Suppl. Figure S4).

Whole-brain CCEP analyses further revealed an interaction between response type and connectivity direction. Specifically, SM sites exhibited stronger outflow than inflow connectivity (LMM: n_conn_=3102, ΔF1=0.042, t=6.16, CI=[0.03, 0.06], p-adj.=3.6e-09), whereas CLX sites showed the opposite pattern, with inflow connectivity exceeding outflow connectivity (LMM: n_conn_=1864, ΔF1=−0.023, t=−2.53, CI=[−0.04, −0.01], p-adj.=0.015) (Fig. 4f, Suppl. Table S2). These findings suggest that SM-responsive sites preferentially broadcast signals, whereas CLX sites may occupy a more integrative role, receiving convergent input from broader brain systems.

### Relationship between lateralization of brain connectivity and bodily responses

As noted above, participants often reported SM responses in the contralateral side of the body while CLX experiences were difficult to lateralize. This prompted us to ask whether the lateralization of brain connectivity is related to the lateralization of stimulation-induced subjective changes. Specifically, we tested whether ipsilateral and contralateral outflow connectivity following single-pulse stimulation could serve as predictors of the laterality of bodily responses evoked by 50 Hz stimulation. Within-subject analyses were not feasible here because the number of observations per condition was insufficient for reliable statistical inference.

As shown in Fig. 4h, the ipsilateral outflow connectivity of the SM but not CLX sites significantly distinguished contralateral from non-lateralized bodily sensations, irrespective of the stimulated hemisphere (Fig. 4h, group-level ANOVA: F=13.42, p=0.0003; post-hoc comparison: ΔF1=−0.04, CI=[−0.06, −0.02], p-adj.=0.0003, also see Suppl. Table S3 for descriptive statistics). Specifically, SM sites that evoked contralateral bodily sensations exhibited stronger ipsilateral outflow connectivity than those evoking non-lateralized responses, suggesting that contralateral bodily sensations are associated with stimulation effects that propagate predominantly within the stimulated hemisphere. This structure–function relationship was specific to SM sites and was not observed for CLX sites (ΔF1=−0.014, p-adj.=0.37, also see Suppl. Table S4). This analysis also echoed our whole-brain fMRI finding (Fig. 4b) by showing that the outflow connectivity was generally higher when the right SM sites were stimulated, comparing to the left, regardless of the laterality of reported bodily responses (Fig. 4h).

### Spatial and phenotypic specificity of cingulo-insular connectivity

**Figure 5.**
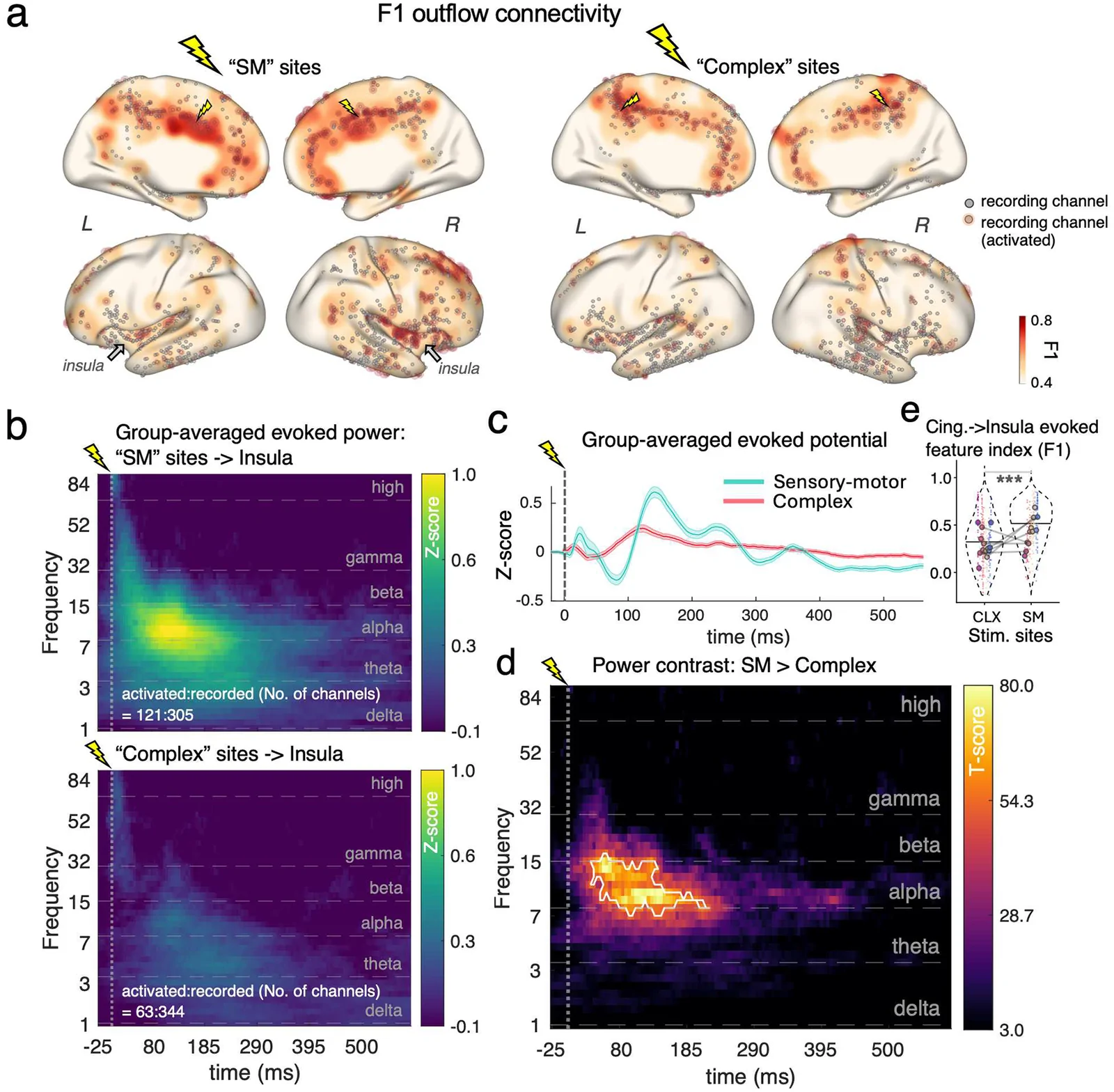
Electrophysiological causal connections between the cingulate/precuneus and insular cortices. **(a)** Whole-brain F1 connectivity measures for all recording channels (grey dots on the surface) are shown after stimulation of SM and CLX sites. Connected sites are marked with an orange circle with a diameter proportional to the F1 value. Each F1 measure for a recording site is averaged across all stimulation sites of both hemispheres in the same category. F1 values of the recording sites are projected to the nearby surface area to show a smoothed regional effect, with a color bar showing weighted-average F1 values, which is corrected for density of local channel coverage. **(b)** Group-averaged evoked spectral power recorded in the insula following stimulation of the “SM” (upper) and “CLX” (lower) cingulate/precuneus sites. The total numbers of sites activated and recorded are noted (see Method for activation detection algorithm). The color bars indicate the z-scored power values. **(c)** Group-averaged evoked potentials in the time domain. **(d)** Contrasted power spectrograms of the cingulate/precuneus -> insula evoked responses between the “SM” and “Complex” stimulating sites. The white contour in the spectrogram indicates a significant cluster (p_cluster < 0.05), corrected using cluster-based permutation. The color bar indicates the T-score. (e) Within-subject comparisons of the cingulate/precuneus -> insula F1 index between the “SM” and “CLX” sites. A mixed linear model, corrected for inter-individual differences, revealed significant differences between the two groups.

When comparing whole-brain connectivity profiles between SM and CLX sites, connections to the insular cortex emerged as one of the most prominent differences (Fig. 5a), motivating a focused analysis of cingulo-insular connectivity. This focus was further supported by two other factors: i) prior neuroimaging studies have consistently linked the cingulate and insula, and ii) our dataset afforded greater simultaneous coverage of these two regions within the same subjects than of any other region pair. We emphasize that this focus does not reflect an a priori assumption that the cingulate-insular pathway is the sole mediator of the subjective effects we report. Rather, we treat the cingulo-insular relationship as a tractable model system for understanding the broader pattern of cross-regional interactions through which localized cingulate stimulation gives rise to conscious bodily experience.

Focusing on the cingulo-insular pathway, group-averaged evoked potentials exhibited significant differences in both the time and time-frequency domains (Fig. 5b–d). Formal statistical inference using a cluster-based permutation test highlighted a significant cluster spanning the 50~230 ms time window (Fig. 5d). Feature extraction, designed to capture this multidimensional evoked effect, aligned well with the signal-based results. Specifically, within-subject comparisons of the strength of evoked responses (i.e., the F1 component) revealed significantly stronger outflow connectivity from SM sites to the insula compared to that from CLX sites to the insula (Fig. 5f; LMM: n_conn_=483, n-sbj.=18, ΔF1=0.11, t=4.46, CI=[0.06, 0.16], p-adj.=2.1e-05), while the reverse connectivity—from the insula to the CC/PCu—did not significantly differ between SM and CLX sites (LMM: n-obs.=472, n-sbj.=20, ΔF1=0.03, t=1.44, CI=[−0.01, 0.09], p-adj.=0.15) (see Suppl. Table S5).

**Figure 6.**
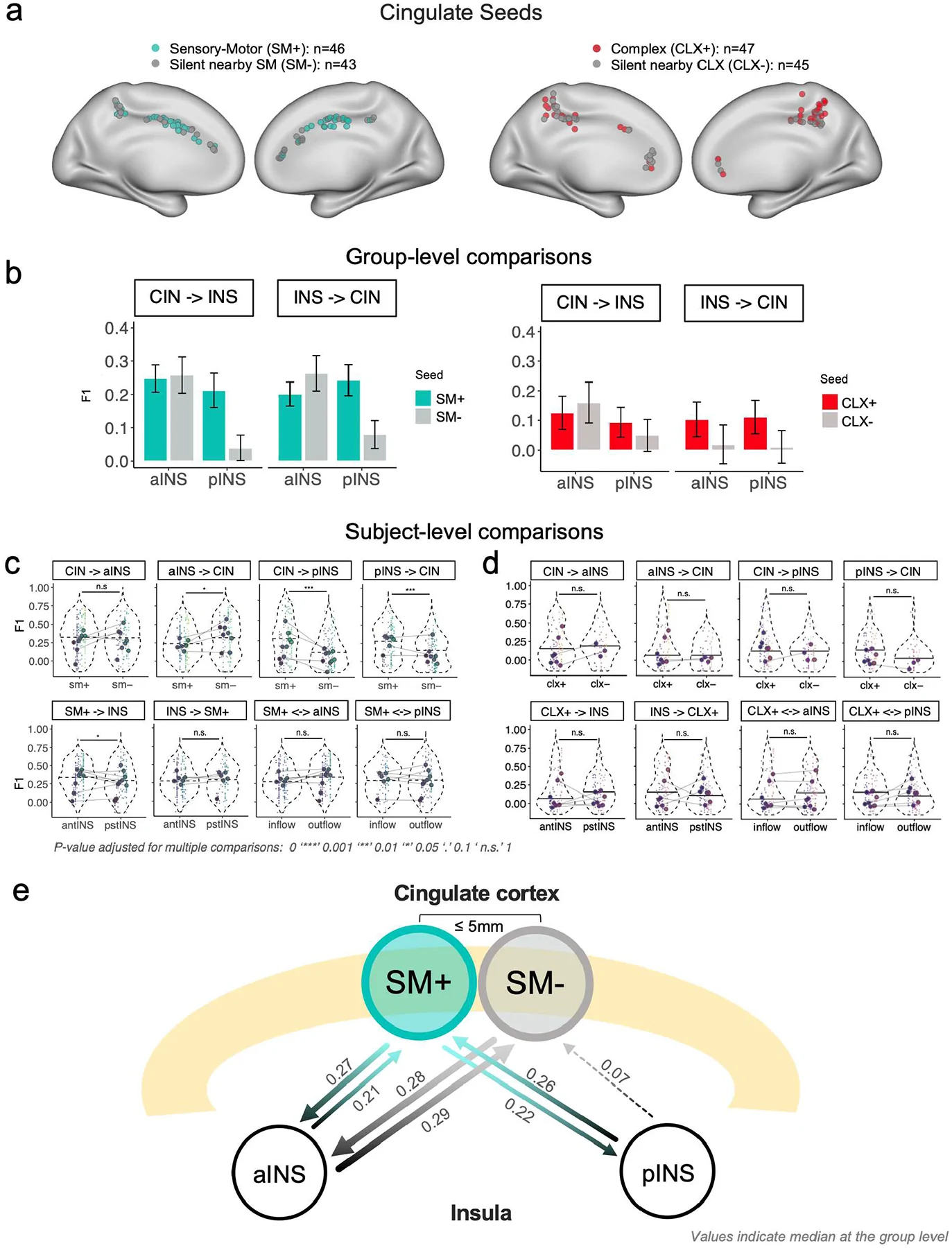
Cingulo-insular local connectivity underlying differential subjective responses: **(a)** Spatial localization of the cingulate seeds whose stimulation caused sensory-motor (SM+) or complex (CLX+) symptoms. Nearby silent electrodes (within 5mm distance) next to these sites (respectively termed as SM−, CLX−) were identified for subsequent connectivity analysis using the measure of F1 (see text for details). Total number of electrode contacts are listed for each category. **(b)** Strength of cingulo-insular F1 connectivity is varied in different conditions, such as different cingulate sites, insula sites, outflow or inflow directions, and responsive or non-responsive sites. Bar lengths of the bar plots indicate the mean while the error bars show two standard errors deviating from the mean. **(c,d)** Post-hoc comparisons of cingulo-insular F1 connectivity in different conditions established in mixed linear models. Big dots on the violin plots show subject-averaged F1 strength, each for one subject in a different color. The thin grey line going through each big dot indicates one standard error deviating from the subject mean. Small dots aligned with the subject-mean dot show F1 of all connections for this subject of this condition. For subjects that have more than 5 data points in both conditions, a grey line across conditions was plotted to show a trend. A dashed line on the violin plot shows the group median. Significance codes are shown for each comparisons, and the p values were adjusted for multiple comparisons. **(e)** Summary of the cingulo-insular causal connectivity in a diagram. The thicker line show stronger connectivity. While a solid line shows strong evidence of connectivity, a dashed line shows weak connectivity. Direction of connectivity is indicated with an arrow.

Because SM and CLX sites occupy distinct cortical locations, differences in their connectivity profiles may simply reflect regional variations in anatomical connectivity rather than the local circuitry underlying the elicited experiences. To minimize this anatomical confound, we compared each responsive site with a nearby non-responsive (“silent”) site within the same participant (≤5 mm away, measured in the normalized FS_LR surface template), respectively for the SM and CLX categories (Fig. 6a). This allowed us to categorize the CC/PCu sites into four roughly equal-sized groups (range = 43–47 sites per group): responsive SM sites (SM+) versus their nearby silent sites (SM−), and responsive CLX sites (CLX+) versus their nearby silent sites (CLX−).

Crucially, we found that cingulo-insular connectivity significantly differentiated responsive sites from their immediately adjacent silent neighbors (LMM: n_conn_=486, n-sbj.=15, ΔF1=−0.07, t=−2.50, p=0.02 for the SM+ vs. SM− comparison). Thus, among neighboring CC/PCu neuronal populations, only those exhibiting direct electrophysiological connectivity with the insula elicited bodily experiences upon stimulation. Adjacent populations, millimeters away, that lack this specific electrophysiological connection remain silent when stimulated. In contrast, although lateral frontal cortex (LFC) connectivity also differed between SM and CLX sites (Figure 5a), it failed to distinguish responsive sites from their adjacent silent neighbors (Suppl. Figure S5), highlighting the selective involvement of local cingulo-insular mesoscale circuitry in induced subjective experiences.

### Directional and subregional insular differences across SM sites

Given the distinct functional roles of the anterior and posterior insula in salience detection and bodily representation^38^, together with the relatively dense insular electrode coverage across participants, we next examined cingulo-insular connectivity in greater detail. Insular recording sites were classified into anterior (aINS) and posterior (pINS) subdivisions using the central insular sulcus as the anatomical landmark. This relatively coarse anatomical classification was chosen to capture the major functional distinction between the aINS and pINS while maintaining sufficient statistical power.

Controlling for causal direction and seed category, we observed a highly significant interaction between the responsiveness of a site within the CC/PCu regions (phenomenological outcome) and the strength of their connectivity with posterior vs anterior insular subregions (anatomical target) (LMM: df=1191, ΔF1=0.14, t=5.64, CI=[0.09, 0.18], p=2.11e-08; Fig. 6b,c), indicating that the subdivision of insular connectivity plays a key role in differentiating responsive sites and their adjacent silent non-responsive sites. Specifically, when predicting whether stimulation of a CC/PCu site would elicit a subjective response, connectivity with the pINS, but not the aINS, emerged as a critical factor. Connectivity with pINS (ANOVA: df=538, ΔF1=0.11, t=6.41, CI=[0.08,0.15], p=1.44e-10), but not aINS (df=649, ΔF1=0.02, t=−1.29, CI=[−0.06,0.01], p=0.199) showed a highly significant main effect in distinguishing responsive from silent sites (Fig. 6b). Moreover, the combination of aINS and pINS inputs significantly differentiated SM and CLX responses from silent neighbors (group-level three-way interaction: responsiveness × insula subregion × response type; df=564, ΔF1=0.18, t=2.24, CI=[0.02,0.33], p=0.025, also see Suppl. Table S6, S7).

To construct a precise region-to-region connectivity map, we focused our pair-wise analyses on the SM-insula network (Fig. 6c). CLX sites were excluded for two reasons: the heterogeneity of CLX responses precluded meaningful statistical inference; and averaging across “CLX” subcategories proved insufficiently sensitive to distinguish the conditions of interest (Fig. 6d). Pairwise within-subject LMM comparisons revealed a striking asymmetry in the connectivity profiles of responsive SM+ sites relative to their neighboring silent SM− sites: SM+ sites maintained strong bilateral connectivity with both aINS and pINS, whereas SM− sites were selectively disconnected from pINS while retaining, or even exceeding, their connectivity with aINS (see full post-hoc comparisons in Suppl. Table S8 & S9). Together, these findings highlight directional and subregional differences in insular connectivity that distinguish responsive from silent sites, identifying pINS input as a key feature of functionally engaged CC/PCu circuitry (Fig. 6e).

## Discussion

Our findings reveal a millimeter-scale functional mosaic within the CC/PCu cortex, where neighboring neuronal populations can engage distinct circuits and produce dissociable subjective bodily experiences. This mosaic is organized not by gross anatomy alone, but by selective connectivity fingerprints, most critically, coupling with the posterior insula. In other words, fine-grained local architecture is embedded within large-scale distributed networks, and the connectivity motif of a stimulated site determines whether local perturbation lead to a change in conscious bodily experience.

Our stimulation mapping confirmed a relationship between the midcingulate cortex and bodily experiences, yet the organization of these subjective representations was markedly different from the canonical somatotopy of primary sensorimotor cortices – as also described by others^23,39^. Although many midcingulate stimulation induced responses were localized to specific body parts, nearly half were non-lateralized and involved whole-body or axial sensations. Rather than reflecting an orderly body map within the CC/PCu cortex, our findings suggest a more heterogeneous representational architecture in which discrete body-part representations coexist with more integrated encodings of whole-body state and action readiness. This organization is well suited to the midcingulate’s proposed role in Action Mode Network (AMN), i.e., coordinating motivated action and interoceptive regulation across the body^30^.

The finding that mid-cingulate sites belonging to AMN had the highest responsiveness (0.68) while sites of default mode network (DMN) had the lowest (0.09) is consistent with the extant literature supporting its role in higher order memory-based and integrative autobiographical processing^8,34^. One possible interpretation is that DMN activity reflects integrative or predictive functions that are not easily perturbed into discrete phenomenological experiences by localized stimulation, whereas AMN sites may be more directly coupled to effector and sensory systems suggested by our prior experimental data^34^.

An important finding in our study was the distinction between simple and complex symptoms caused by the stimulation of distinct, and at times the adjacent CC/PCu complex. While stimulation of sites in the mid-cingulate region predominantly elicited simple discrete, bodily sensations, stimulation of adjacent sites or the ones in the anterior precuneus gave rise to a more diffuse, integrated bodily states. This phenomenological distinction was mirrored in the causal connectivity profiles of the two site types. While the SM responsive sites exhibited stronger outgoing (divergent) connections broadcasting to distributed sensory and sensorimotor regions, the CLX sites showed the opposite pattern, with convergent inflow from multiple systems predominating. This dissociation suggests a functional hierarchy within the mosaic CC/PCu architecture: SM sites may implement or modulate specific bodily representations and relay them outward, whereas CLX sites occupy a more integrative position, combining signals across multiple systems to construct higher-order representations of bodily state and self.

A particularly important finding pertains to the differential roles of insular subregions in gating bodily experience. Connectivity with the pINS, but not the aINS, distinguished responsive from silent CC/PCu sites, suggesting that it is specifically the grounded bodily representation carried by the posterior insula - rather than the affective and interoceptive integration associated with the anterior insula - that is critical for cingulate-mediated subjective experience. Our findings suggest that connections with the posterior region of the insula may be important for cingulate perturbations to produce changes in conscious bodily experience. This is further corroborated by stimulation studies showing that, unlike posterior insular stimulation, anterior insular stimulation does not reliably alter subjective state despite evoking strong prefrontal and CC/PCu responses and that the aINS–CC/PCu axis may serve a regulatory or predictive top down role rather than a direct phenomenological one – as we have detailed recently^38^.

Both the open neuroimaging dataset and our electrophysiological stimulation data revealed a robust rightward bias in the network associated with the midcingulate. Although hemispheric asymmetry is a fundamental feature of nervous system organization^40,41^, large-scale intrinsic networks are often treated as bilaterally symmetric. Our findings challenge this assumption by showing preferential engagement of right-hemisphere circuitry in both functional and causal connectivity analyses, even though stimulation in either hemisphere could evoke bodily experiences. Our results are consistent with previous neuroimaging studies showing right-lateralized organization of brain systems involved in interoceptive and self-related processing^42,43^ and provide potential mechanistic framework for understanding the right dominance of hemispatial neglect. Notably, this asymmetry was not explained by the often-cited right dominance of the aINS^44^, as SM–aINS connectivity showed no significant lateralization in either direction (aINS→SM, p = 0.91; SM→aINS, p = 0.15). Instead, the results suggest a broader right-biased network architecture, potentially involving the AMN^30^, of which aINS lateralization may represent one manifestation.

Several key features of our methodological approach deserve explicit consideration. First, our observations were obtained from neurosurgical participants with focal epilepsy, which provides a rare opportunity to combine causal perturbation with first-person phenomenological report in the human brain, a combination unavailable in healthy volunteer populations. Several factors support the generalizability of our findings: most sampled CC/PCu sites were radiologically normal and outside the seizure-onset zone, and our prior work indicates that such tissue shows physiologically meaningful stimulation responses indistinguishable from those in non-epileptic tissue when seizures or pathological discharges are absent. Second, sEEG electrode placement was determined by clinical considerations rather than experimental design, resulting in sparse and uneven coverage across participants. This clinical constraint is, however, offset by the unparalleled spatial resolution and individual-level anatomical precision that intracranial recordings afford, a resolution inaccessible to any non-invasive method. Third, some co-registration uncertainty is inherent to electrode localization, but this is unlikely to account for the observed mosaic organization, since responsive and silent sites were compared within the same individuals at millimeter-scale spatial proximity (Suppl. Figure S3). Fourth, CCEP-derived connectivity reflects perturbational rather than resting-state or true functional connectivity during a task in real life. It characterizes pathways through which stimulation-evoked activity propagates, offering a causally grounded measure that complements but is distinct from structural connectivity or spontaneous functional coupling. Finally, subjective reports are intrinsically variable and depend on patients' attention, verbal capacity, and willingness to describe internal states - an irreducible feature of any study that takes first-person phenomenology seriously as a scientific dependent variable. This variability is particularly relevant for the CLX category, whose internal structure warrants further resolution in future work using more structured phenotyping of stimulation-induced bodily experiences.

Our approach also differs from prior cingulate stimulation studies in several deliberate and consequential respects, most notably from Caruana et al. (2018)^23^. We used a pulse-width of 0.2 ms compared to 1 ms in that study - a fivefold difference that substantially reduces total charge delivery per pulse and restricts the effective stimulation volume by limiting current spread to adjacent white matter fibers, thereby enhancing the spatial specificity of the functional mapping. Our stimulation trains were also shorter, further reducing total charge per trial. Additionally, we reviewed the position of the electrodes in native anatomical slices and carefully excluded stimulations centered in the white matter, or in dorsal cingulate sites extending into the medial frontal cortex adjacent to the SMA and pre-SMA — regions independently known to generate motor and behavioral responses — or in the most ventral posteromedial cortical regions abutting the visual cortices and bypassing visual tracts - ensuring that our mapping reflects stimulation of CC/PCu tissue proper. Beyond spatial precision, our protocol required all reported subjective responses to satisfy four validation criteria: absence during after-discharges or seizures, reproducibility across repeated stimulations of the same site, a clear dose-response relationship, and absence during sham trials. These criteria, not applied in prior studies, provide a rigorous causal standard for attributing responses to focal cortical activation rather than to placebo effects, spontaneous experiences, or subclinical ictal phenomena. Together, these methodological choices reflect a deliberate prioritization of spatial precision, causal validity, and phenomenological rigor over sheer sample size - a trade-off that we believe is well justified given the questions this study set out to address.

We believe our findings may have implications for circuit-based neuromodulation. The CC/PCu cortex is an emerging stimulation target for treatment-resistant psychiatric disorders, yet clinical outcomes remain variable^45,46^. Our results suggest that part of this variability may arise because neighboring CC/PCu sites, even when located within the same gross anatomical region or imaging-defined network, can engage distinct mesoscale electrophysiological circuits and produce markedly different subjective effects. This supports the need for individualized, circuit-informed targeting approaches that account for millimeter-scale connectivity profiles rather than anatomical location alone. More broadly, these findings show that local neural activation is not sufficient to generate conscious bodily experience; a stimulated population becomes subjectively reportable only when embedded within the appropriate connectivity architecture. Thus, millimeter-scale functional mosaics are nested within large-scale brain networks, and their connectivity—not anatomy alone—determines whether local stimulation becomes conscious bodily experience.

## Methods

### Participants

Data reported in this study were obtained from a large cohort of 63 participants undergoing direct electrical stimulation of the cingulate/precuneus (CC/PCu) sites over the last 18 years of our practice at Stanford University Medical Center. The cohort underwent routine clinical intracranial electrical stimulation (iES) for functional mapping prior to surgical resection. This dataset was used to characterize the anatomical distribution and phenomenology of subjective experiences elicited by cingulate stimulation. In total, 660 bipolar sites distributed across the cingulate cortex and adjacent posteromedial cortices were stimulated for this procedure.

Among them, the 28 most recently recruited participants underwent repeated single-pulse electrical stimulation (SPES) for characterizing causal electrophysiological connectivity throughout the human brain. These participants contributed 71.43 ± 24.52 stimulation channels, 147.04 ± 10.51 recording channels, and 10,768.04 ± 4,606.09 directed stimulation–recording, and in total 301,505 stimulation-recording pairs spanning the whole brain at the group level.

For both datasets, electrode implantation was determined exclusively by clinical considerations and was independent of the present study. All participants provided written informed consent before participation. Experimental procedures were approved by the Stanford University Institutional Review Board and were conducted in accordance with institutional guidelines.

### Electrode localization

A thin-cut head CT acquired after electrode implantation was coregistered to each participant’s preoperative 3 T MRI to verify electrode trajectories. Cortical surface reconstruction was generated from the T1-weighted MRI using the recon-all command in FreeSurfer v7.3.2^47^. The post-implant CT was then coregistered to the pre-implant MRI using FLIRT from the Oxford Centre for Functional MRI of the Brain Software Library (FSL)^48,49^. Individual electrodes were manually identified on the T1-registered CT images using BioImage Suite. Electrode coordinates in FreeSurfer surface space, voxel space, and MNI305 space were automatically extracted using the iElVis toolbox^50^. For group-level visualization, each participant’s electrode coordinates were registered to the FS_LR standard surface template, which provides homologous left and right hemispheres, using Connectome Workbench commands (https://www.humanconnectome.org/software/connectome-workbench).

All electrode contact locations were manually assigned to anatomical regions of interest by an experienced neurologist on the team (J.P.), based on each participant’s individual brain morphology and anatomical landmarks. This process involved careful inspection of the fused CT–MRI images in axial, sagittal, and coronal planes, with attention to the implanted contacts and surrounding anatomical structures. Contacts located entirely or partially outside gray matter were excluded from further analysis. Importantly, regions of interest were not assigned in standard atlas space but were identified in each participant’s own high-resolution T1-weighted MRI to maximize the anatomical precision of electrode localization.

### Clinical 50Hz intracranial electrical stimulation

IES was performed as part of routine functional mapping before neurosurgical resection. Bipolar stimulation was delivered between adjacent electrode contacts using a Nihon Kohden Cortical Stimulator (MS-120BK-EEG) connected through a PE-210AK stimulator switchbox.

The clinician performing the procedure determined stimulation parameters according to clinical requirements. Stimulation typically consisted of alternating square-wave pulses (0.2ms pulse-width) delivered at 50 Hz for 1–2s with current amplitudes below the threshold for producing after-discharges (typically 2–10 mA). Individual stimulation trials varied slightly according to clinical necessity, but stimulation parameters were digitally logged for every trial together with contemporaneous bedside notes. Repeated trials with sham stimulations and trials with varied amplitudes and durations were always applied to validate the categorization of a responsive or silent site.

Immediately after stimulation, participants were encouraged to describe any subjective experiences as they occurred during the stimulation. Observable behavioral responses, including involuntary movements or changes in facial expression, were simultaneously documented by the clinical team. Sham stimulation trials were interleaved when clinically feasible to assess response reliability and minimize expectation effects.

Video recordings synchronized with stimulation timing were reviewed offline. Subjective reports were independently transcribed and compared against bedside notes. Initial interpretation of each response was performed by the examiner who conducted the stimulation, after which classifications were independently reviewed by at least two additional raters who were blinded to the study hypotheses. Discrepancies were resolved by consensus.

### Population-level functional connectivity analysis

To characterize the large-scale functional organization surrounding stimulation-responsive cingulate sites, we analyzed resting-state functional connectivity using the Human Connectome Project (HCP) V210 preprocessed dataset^35^. Seed-based functional connectivity maps were generated for vertices spanning the cingulate cortex and adjacent posteromedial cortex. Pearson correlation coefficients between each seed vertex and every cortical vertex were calculated using resting-state BOLD time series which are preprocessed by the data distributor. See details of the preprocessing pipeline in their previous publication^35^.

To statistically assess the lateralization of the cingulate cortex in terms of its network associations, we compared functional connectivity (FC) between homologous vertices in the left and right hemispheres. Less positive FC values (Pearson *r* < 0.3) were replaced with zeros to avoid interpretational ambiguities due to sign differences, thereby excluding anti-correlated connections from the analysis. A one-sample t-test was performed on the distribution of left–right FC differences for each vertex within the cingulate cortex and the posteromedial cortex (PMC; n = 2,902). Vertices located on the medial wall and within the same cingulate parcellation were masked out to eliminate self-connections and irrelevant contributions, resulting in df ≥ 940. Bonferroni correction was applied to control for Type I error inflation from multiple comparisons. As a result, each cingulate/PMC vertex was assigned a significance level (p-FWE < 0.001) indicating the degree of lateralization in its whole-brain FC pattern.

### Repeated single-pulse electrical stimulation

Causal electrophysiological connectivity was quantified using repeated single-pulse electrical stimulation (rSPES), alternatively called cerebro-cerebral evoked potential (CCEP). In this stimulation protocol, biphasic square-wave single pulses (6 mA, 0.2 ms pulse width; and the amplitude was reduced to 4 mA close to an epileptic zone) were delivered through bipolar pairs of adjacent intracranial electrode contacts, while electrophysiological activity was simultaneously recorded from all remaining implanted contacts. Each stimulation consisted of approximately 42 ± 2 repeated pulses separated by a 2-second interval, together delivered over a period of approximately 1.5 min.

To ensure reliable estimation of physiological connectivity, stimulation pairs located outside grey matter, spanning different anatomical regions, or situated within 5 mm of the recording contacts were excluded from subsequent analyses. Stimulation artefacts confined to the immediate post-stimulation period (<10 ms) were excluded from the formal analyses.

### iEEG data acquisition and preprocessing

Simultaneous intracranial electrophysiological recordings during rSPES was collected using the Nihon Kohden recording system with a sampling rate of 1,000 Hz. The iEEG data were preprocessed using the pipeline described previously^36^. Briefly, signals were notch-filtered at 60, 120 and 180 Hz to remove line noise and harmonics. Channels containing excessive noise or pathological activity and trials contaminated by recording artefacts were excluded before further analyses. Because SPES was performed using bipolar stimulation, recordings were re-referenced using bipolar subtractions.

Signals were epoched relative to stimulation onset, baseline corrected using the pre-stimulation period and normalized to the baseline standard deviation. To characterize stimulation-evoked responses in both the temporal and spectral domains, bipolar voltage traces were decomposed using Morlet wavelets spanning frequencies from 1 to 256 Hz. Spectral power and inter-trial phase coherence (ITPC) were calculated for each stimulation-recording pair and averaged across repeated stimulation trials.

### Feature-based causal electrophysiological connectivity

Directed electrophysiological connectivity was quantified using the feature-based stimulation-response framework developed in our previous study^36^. Conventional time-domain quantification of CCEPs relies on identifying characteristic waveform peaks (e.g., the N1), but this approach can be challenging for responses with heterogeneous morphologies, variable latencies, or leaking stimulation artefact. To overcome these limitations, stimulation-evoked responses were jointly represented by their temporal waveform, spectral power, and inter-trial phase coherence. These multidimensional responses were analyzed using our previously developed semi-supervised machine-learning framework, which classified recording channels as activated or non-activated for each stimulation site, reflecting physiologically connected and disconnected stimulation–response pairs, respectively, and identified reproducible physiological features of stimulation-evoked activity. Here, we focused on the first feature (F1), an early (approximately 10–60 ms), sharply peaked, and highly phase-locked stimulation-evoked response pattern that closely corresponds to the conventional N1 component while providing a more robust representation across heterogeneous response morphologies. Because the F1 feature score quantifies the expression strength of this canonical early response pattern, it was used as the measure of causal connectivity strength throughout this study.

### Whole-brain causal connectivity analysis

Whole-brain causal connectivity profiles were constructed for each responsive cingulate stimulation site by extracting F1 connectivity scores to every available recording site throughout the brain. Connectivity was analyzed at the level of individual stimulation-recording pairs while accounting for repeated observations per region per participant.

To examine hemispheric asymmetry, recording sites were assigned to either the ipsilateral or contralateral hemisphere relative to the stimulated cingulate site. Mean F1 connectivity was then compared across hemispheres separately for sensorimotor (SM) and complex bodily (CLX) stimulation sites. A nested random effect of brain region within participant was included to account for within-participant, within-region variability during left–right hemisphere comparisons.

To characterize the directional organization of the cingulate network, outflow and inflow connectivity were analyzed separately. Outflow connectivity quantified the strength of causal influences from CC/PCu stimulation sites to recording sites elsewhere in the brain, whereas inflow connectivity quantified causal influences from stimulation of other brain regions onto recording sites within the CC/PCu. To compare the inflow and outflow connectivity, outgoing and incoming connectivity strengths were compared within CC/PCu sites using linear mixed-effects models, with “connectivity direction” as a fixed effect and distal brain regions nested within participants included as a random effect.

### CC/PCu-insula connectivity analysis

For each CC/PCu -insula connection, trial-averaged evoked spectrograms were compared directly in the time-frequency domain. Differences in stimulation-evoked time-frequency responses were evaluated using cluster-based permutation tests, which controlled the family-wise error rate across both temporal and spectral dimensions. It was conducted using the toolbox of MNE-python (https://mne.tools/stable/index.html).

To provide a quantitative measure of these differences, F1 connectivity values were extracted for every CC/PCu -insula connection. Connectivity strength was compared between sensorimotor and complex bodily stimulation sites separately for outflow (CC/PCu - to-insula) and inflow (insula-to- CC/PCu) directions.

To dissociate the effects of mesoscale electrophysiological circuit and macroscale anatomical landscape, responsive sites were compared with anatomically adjacent non-responsive (“silent”) sites located within a 5-mm radius (distance was normalized in the FS_LR standard surface template). Connectivity profiles of responsive and nearby silent sites were compared separately for the sensorimotor and complex bodily groups.

### Statistical analysis

Statistical analyses were performed using MATLAB and R. Unless otherwise specified, electrophysiological connectivity analyses were conducted using linear mixed-effects models (LMMs) to account for repeated observations obtained from the same regions in the same participants. When whole-brain CCEP was involved, a nested random effect of regions within participants was used, otherwise, participant identity was always included as a random intercept, whereas fixed effects varied according to the specific analysis and included factors such as stimulation site category (sensorimotor versus complex bodily), hemisphere, connectivity direction (incoming versus outgoing), response laterality, responsiveness (responsive versus silent).

Estimated marginal means were used for post hoc comparisons, and *p* values were adjusted for multiple comparisons where appropriate using the Holm-Bonferroni procedure. Model estimates are reported together with 95% confidence intervals.

For categorical analyses, χ^2^ tests were used to compare response frequencies across anatomical subdivisions and hemispheres. Group-level comparisons involving multiple factors were evaluated using analysis of variance (ANOVA) where appropriate. All statistical tests were two-sided, and statistical significance was defined as P < 0.05 after correction for multiple comparisons unless otherwise stated.

## Supplementary Material

This is a list of supplementary files associated with this preprint. Click to download.

- SIHCPFCrightseeds.mp4
- SIHCPFCleftseeds.mp4
- SupplementaryMaterials.pdf

## Figures and Tables

**Figure 2.**
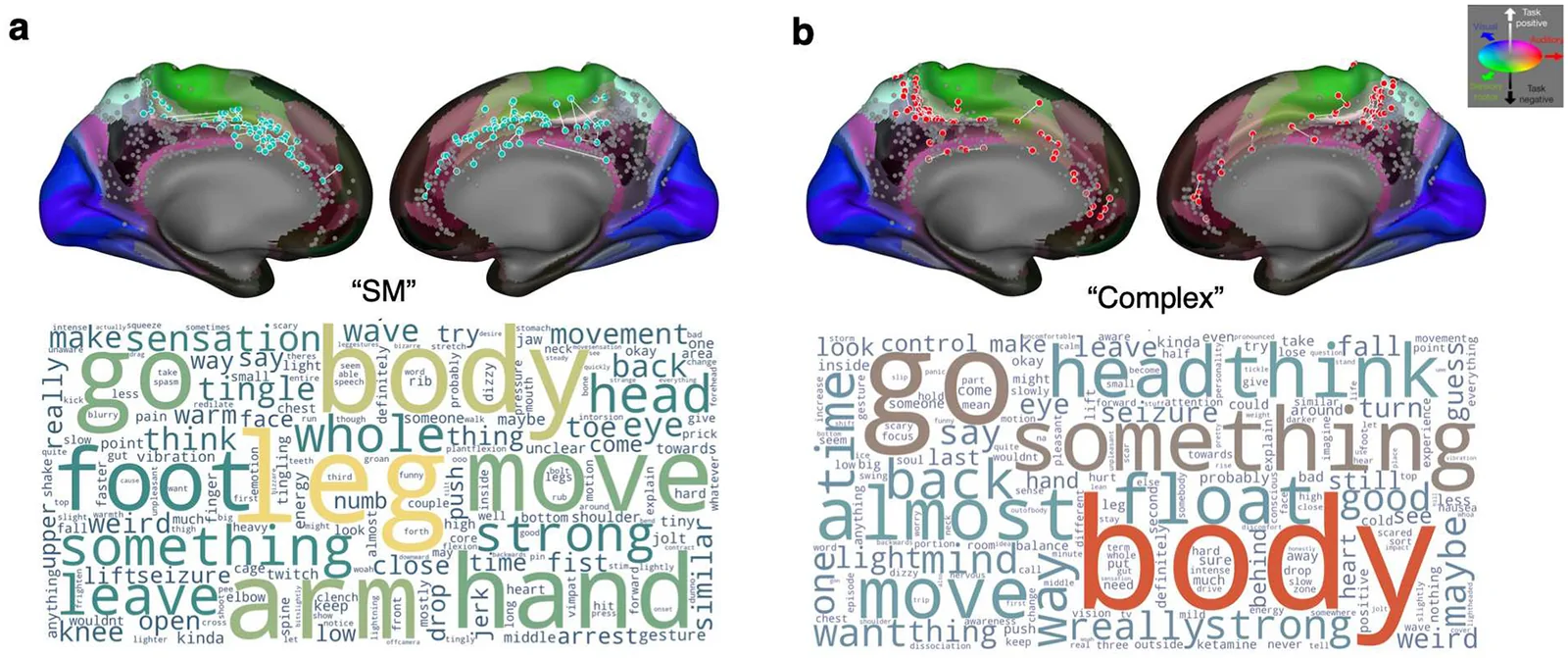
Subjective Changes Associated with Electrical Stimulation of the Cingulate/Precuneus Cortex. (a) Stimulation locations (turquoise) for “SM” subjective changes are superimposed on the Glasser parcellation^35^. A word cloud of all reported subjective changes in this behavioral category, as narrated by the participants, is shown below. The size of each word is proportional to the frequency with which it was reported at the group level. (b) Stimulation locations (red) for “Complex” subjective changes are visualized using the same scheme as in (a). The behavioral correspondence of the Glasser parcellation, primarily determined by neuroimaging studies, is shown in the upper right. We show only SM and Complex categories as we had the largest number of observations for these categories. Word counts of the subjective reports are presented in Suppl. Figure S1.

## Data Availability

Electrode-level information, including spatial localizations and response classifications following 50-Hz stimulation, is publicly available through Zenodo at https://doi.org/10.5281/zenodo.21536139. Source data for reproducing the Figures 4h, 6b are provided in the Supplementary Materials. Additional non-identifiable data are available from the corresponding author upon reasonable request.

## Code availability statement:

Example custom code used to generate the key results reported in this study is publicly available in the Zenodo repository at 10.5281/zenodo.21536139.
